# MRI tracking of human Wharton’s jelly stem cells seeded onto acellular dermal matrix labeled with superparamagnetic iron oxide nanoparticles in burn wounds

**DOI:** 10.1093/burnst/tkac018

**Published:** 2022-11-11

**Authors:** Davood Mehrabani, Mehra Nazempour, Rouhollah Mehdinavaz-Aghdam, Seyedeh-Sara Hashemi, Reza Jalli, Mahdi Saeedi Moghadam, Shahrokh Zare, Iman Jamhiri, Javad Moayedi, Feridoun Karimi-Busheri

**Affiliations:** Stem Cell Technology Research Center, Shiraz University of Medical Sciences, Shiraz, Fars T6G 2E1, 71348-14336, Iran; Burn and Wound Healing Research Center, Shiraz University of Medical Sciences, Shiraz, Fars 71987-74731, Iran; Comparative and Experimental Medicine Center, Shiraz University of Medical Sciences, Shiraz, Fars 71348-14336, Iran; Li Ka Shing Center for Health Research and Innovation, University of Alberta, Edmonton, Alberta, Canada; Department of Biomedical and Tissue Engineering, Science and Research Branch, Islamic Azad University, Tehran, Iran; School of Metallurgy and Materials Engineering, College of Engineering, University of Tehran, Tehran 1417614411, Iran; Burn and Wound Healing Research Center, Shiraz University of Medical Sciences, Shiraz, Fars 71987-74731, Iran; Medical Imaging Research Center, Department of Radiology, Shiraz University of Medical Sciences, Shiraz, Fars 71348-14336, Iran; Medical Imaging Research Center, Department of Radiology, Shiraz University of Medical Sciences, Shiraz, Fars 71348-14336, Iran; Stem Cell Technology Research Center, Shiraz University of Medical Sciences, Shiraz, Fars T6G 2E1, 71348-14336, Iran; Department of Biochemistry, School of Biotechnology and Agriculture, Shiraz Branch, Islamic Azad University, Shiraz, Fars 71987-74731, Iran; Stem Cell Technology Research Center, Shiraz University of Medical Sciences, Shiraz, Fars T6G 2E1, 71348-14336, Iran; Comparative and Experimental Medicine Center, Shiraz University of Medical Sciences, Shiraz, Fars 71348-14336, Iran; Department of Oncology, Faculty of Medicine, University of Alberta, Edmonton, Alberta, T6G 1Z2, Canada

**Keywords:** Wharton’s jelly stem cells, Superparamagnetic iron oxide nanoparticles, Acellular dermal matrix, Magnetic resonance imaging, Healing, Burn

## Abstract

**Background:**

*In vivo* cell tracking after transplantation in regenerative medicine remains an unmet challenge and limits current understanding of the wound healing mechanism through cell-based therapies. This study investigated tracking of human Wharton’s jelly stem cells (hWJSCs) seeded onto an acellular dermal matrix (ADM) and labeled with superparamagnetic iron oxide nanoparticles (SPIONs) by magnetic resonance imaging (MRI) in burn injury.

**Method:**

The hWJSCs were characterized and assessed for growth kinetics. A total of 30 rats were enrolled in three equal groups. Group 1 underwent scald burn injury left without treatment, the group 2 was treated by an ADM that was prepared from cosmetic surgery skin samples and the group 3 received hWJSCs labeled with SPIONs seeded onto an ADM. Tensile strength was evaluated before and after interventions, real time PCR assessed apoptosis, and Prussian blue staining, scanning electron microscopy (SEM) and MRI were used for the tracking of labeled cells.

**Results:**

The hWJSCs exhibited mesenchymal stem cell properties. Population doubling time was 40.1 hours. SPIONs did not show any toxic effect. The hWJSCs seeded onto an ADM decreased Bax and increased Bcl-2 gene expression. Internalization of SPIONs within hWJSCs was confirmed by Prussian blue staining, SEM and MRI until day 21. There was a significant difference between the Young’s moduli of normal skin and the group receiving hWJSCs seeded onto an ADM. Histological observations and SEM imaging confirmed that MRI is an accurate method to track SPION-labeled hWJSCs *in vivo*.

**Conclusions:**

This study showed that SPION labeling coupled with MRI can be used to further understand the fate of stem cells after transplantation in a burn model.

## Highlights

• Human Wharton’s jelly stem cells can be easily labeled with superparamagnetic iron oxide nanoparticles and seeded onto an acellular dermal matrix.

• The labeled stem cells when seeded onto an acellular dermal matrix in burn wounds can have a curative role in the absence of any complications in burn wounds.

• Magnetic resonance imaging is an accurate and non-invasive method to track labeled stem cells in the site of tissue injury and assess treatment outcome.

## Background

Burn injuries are difficult to manage due to the risk of infection and the loss of skin integrity that can lead to severe morbidity. They are responsible for an economic health burden, especially in cases of severe burn injuries where the healing process does not start immediately following the injury [1]. Conventional management for severe burn injuries involves the excision of the thermally injured skin and epidermal replacement by autologous split-thickness skin graft harvest from a healthy donor site from the same patient; this treatment modality is lengthy, costly and risky and may lead to further debilitation [2]. Therefore, there is a need for a safe, easy-to-use and effective technique for skin repair. Regenerative medicine by utilization of mesenchymal stem cells (MSCs) was shown to be beneficial in cutaneous wound healing and skin regeneration via acceleration of wound closure, enhancement of re-epithelialization, increase in angiogenesis, modulation of inflammation and regulation of extracellular matrix (ECM) remodeling [3]. MSCs are currently a topic of interest in tissue healing, because they can differentiate into mesodermal, ectodermal and endodermal cells with low immunogenicity and paracrine activities [4].

MSCs have been extracted from different tissue sources such as bone marrow [5], adipose tissue [6], dental pulp [7] and Wharton’s jelly [8]. Wharton’s jelly-like matrix of the umbilical cord is the source of human Wharton’s jelly stem cells (hWJSCs). Due to their high immunomodulatory activity, MSCs are ideal for allogenic use and for making ‘off-the-shelf’ skin substitutes, allowing them to be applied clinically for a broad scope of diseases in regenerative medicine [9,10]. They can be easily cultured, express MSC markers and lack hematopoietic markers [11]. They can have favorable effects on the healing response by regulating the inflammatory response in wounds. However, when MSCs are transplanted locally, no long-lasting retention in sites of injury was noted and few MSCs arrived and engrafted at the injury site, thus leading to a slow recovery [12].

Therefore researchers have investigated ways to increase MSC mobilization and migration into cutaneous wounds such as seeding MSCs onto scaffolds with properties such as porosity, cytocompatibility, biodegradability, surface physicochemical and biomechanical activities, providing a 3D environment to improve cell proliferation and differentiation and finally promote healing of the injured tissue [8,13]. Advances in dermal scaffolds led to the introduction of the popular dermal scaffold material called acellular dermal matrix (ADM) used in esthetic medicine and healing of burn injuries [14–16].

It is always a challenge to track MSCs after transplantation within the body and little information is available after cell transplantation into the body to help investigate the mechanisms of cell behavior and function in tissue repair [17]. Magnetic resonance imaging (MRI) was introduced as a non-invasive method of cell monitoring providing high spatial resolution images in clinics [17]. In MRI, contrast agents consist of paramagnetic or superparamagnetic metal ions that increase the sensitivity of MRI in the detection of many pathological events [18]. Among contrast agents, superparamagnetic iron oxide nanoparticles (SPIONs) are available as biocompatible small crystalline magnetite structures (5–150 nm) and are easily internalized into the cell [18].

SPIONs coated with dextran or carboxy dextran have clinical approval. They have been successfully used in cell labeling that can be tracked and imaged easily in cells by MRI and electron microscopy both *in vitro* and *in vivo* [19]. In MRI, SPIONs are imaged by T2/T2-weighted sequences affecting T2 relaxation and causing enhancement of T1-weighted images [18]. Several researchers have also shown that these nanoparticles do not have any negative effect on cell proliferation, viability, phenotype or functional properties *in vitro* [20] and can even improve the viability and fate of MSCs [21]. There are still inconsistencies that may be due to variation in SPION particle size, surface-coating material, their incubation time, the labeling technique and the MSC source [22] This study has investigated the use of non-invasive MRI in tracking of hWJSCs labeled with SPIONs and seeded onto ADM scaffold in the healing of scald burn injury in an experimental rat model.

## Methods

### Isolation of hWJSCs

Human umbilical cord tissue was used to prepare hWJSCs by exposing the tissue to enzymatic digestion [23]. Three healthy umbilical cord tissues were prepared from volunteers following the signing of a consent letter for the use of human skin samples in the research. The samples were transferred into a falcon tube containing phosphate-buffered saline (PBS: Sigma-Aldrich), penicillin, streptomycin and amphotericin B (100 U/mL, 100 μg/mL and 0.25 μg/mL, respectively; Invitrogen) and were aseptically taken to the Burn and Wound Healing Research Center, Amiralmomenin Hospital, Shiraz University of Medical Sciences, Shiraz, Iran.

Tissue samples were washed three times with Hanks’ balanced salt solution (Invitrogen) and were horizontally chopped into 1 cm pieces and then transferred into a falcon tube containing collagenase type I, collagenase type IV (Thermo Fisher Scientific) and hyaluronidase (Sigma-Aldrich) for enzymatic digestion. They were later put in an incubator with 5% CO_2_ and saturated humidity at 37°C for 45 min to create a dissolved gelatinous mass of Wharton’s jelly. Under a laminar flow hood in aseptic conditions, the gelatinous masses were filtered by an 18-gauge needle syringe to facilitate the release of cells from the masses.

The filtrate was centrifuged for 7 min at 200 g, the supernatant was removed and the cell pellet was carefully re-suspended in 1 mL of a medium containing 90% Dulbecco’s modified Eagle’s medium/F12 (DMEM/F12, Invitrogen) with 10% fetal bovine serum (FBS) (Gibco), 1% nonessential amino acids (Invitrogen), 2 mmol/L L-glutamine (Invitrogen) and 1% penicillin, streptomycin and amphotericin B. The suspended cells were transferred into a culture flask already containing 4 mL of 90% DMEM/F12 with 10% FBS, 1% nonessential amino acids, 2 mmol/L L-glutamine and 1% penicillin, streptomycin and amphotericin B. The viable cells were plated at a density of 1 million cells/cm^2^ in 100-mm cell culture dishes. After 5 days, the medium was changed and subsequent medium replacement happened every 3 days. The culture dishes were put in an incubator with 5% CO_2_ and saturated humidity at 37°C. Cell counting was carried out by trypan blue dye using a Neobar hemocytometer.

### Characterization of hWJSCs

#### Morphological characterization

The hWJSCs were investigated to be morphologically spindle shaped using an inverted microscope [19].

#### Characterization by osteogenic induction

To assess osteogenic differentiation, 5 × 10^4^ hWJSCs were transferred into 6-well plates. At 80% confluence, the medium was replaced with osteogenic medium containing complete culture medium supplemented with 15% FBS, 100 nM dexamethasone (Sigma-Aldrich), 50 μM ascorbic acid (Merck) and 10 mM glycerol 3-phosohate (Merck) for 21 days. Medium change was conducted every 2 days and after 21 days 10% formalin was used for 20 min to fix hWJSCs, and following three washes with deionized water, the cells were stained using 1.4% alizarin red dye dissolved in deionized water at pH of 4.1 (Sigma-Aldrich). Differentiation was assessed by alizarin red staining that is bound to calcium mineralized deposits revealing a red color [19, 24].

#### Characterization by adipogenic induction

In total, 5 × 10^4^ hWJSCs were seeded in 6-well plates containing culture medium. At 80% confluence, the medium was changed to an adipogenic medium consisting of complete culture medium supplemented with 15% FBS, 100 nM dexamethasone, 100 μM ascorbic acid and 200 μM indomethacin (Sigma-Aldrich) for 21 days. Formalin (10%) was used for 20 min to fix the cells and after washing three times with deionized water, fresh 0.5% Oil Red O dye (Sigma-Aldrich) dissolved in 2-propanol solution (Merck, Darmstadt, Germany) for 2 h was used for staining of differentiated hWJSCs. Oil Red O staining reveals red color droplets when adipogenic induction happens [19, 24].

#### Immunophenotypic characterization

The analysis of cluster of differentiation (CD) surface marker was conducted by cell harvesting, washing with cytometer buffer [PBS + 2% bovine serum albumin (BSA; Biological Industries)] at 600 g for 5 min and incubation with specific labeled antibodies in cytometer buffer at 4°C for 20 min. Primary antibodies with fluorophores were utilized against human cell surface CD antigens CD44, CD73, CD90 and CD105 as mesenchymal stem cell markers, CD34 and CD45 as hematopoietic stem cell markers and Notch1. Antibodies used in this study and their sources are listed in Table 1. Each marker was separately evaluated for each sample. Matching isotype antibodies were applied as negative controls. One technical replicate was used for each sample. For viability staining, 7AAD dye was utilized. Data were collected using a FACS Aria II flow cytometer (BD Biosciences) and analyzed using FacsDiva analysis software (BD Biosciences) while gating the live cells using appropriate isotype controls against each fluorophore for determining non-specific binding.

### Growth kinetics

In total, 4 × 10^4^ hWJSCs from passage 3 were seeded in 24-well culture plates and left in an incubator with 5% CO_2_ and saturated humidity at 37°C. Medium change took place every 3 days and cell viability and the population doubling time (PDT) were determined for 7 days using GraphPad Prism (GraphPad Software Inc), a phase contrast microscope, 0.4% trypan blue solution (Biowest) and a Neubauer hemocytometer. Measurements for each sample were repeated three times. Cells were cryopreserved at a density of 1 × 10^6^ cells/mL in 10% dimethyl sulfoxide (DMSO; MP Bio) (v/v), 50% FBS (v/v) and 40% DMEM.

### Cell labeling

A total of 3.5 mg/mL of SPIONs was used to label hWJSCs as described before [19]. These particles consisted of 75–80% (w/w) magnetite in a matrix of dextran (40 000 Da), that were conjugated with OH, NH_2_ and COOH groups for the covalent binding of proteins, antibodies or other molecules (Nanomag magnetic dextran nanoparticle, 130 nm particle size, Micromod Partikel Technologie GmbH, Rostock, Germany). Following the incubation of hWJSCs labeled with SPIONs for 48 h and three washes with PBS to remove excess nanoparticles, the cells were trypsinized. The labeled cells were later centrifuged at 300 g for 5 min and after removal of the supernatant, the cell pellet was carefully re-suspended in 1 mL of the medium to be used for subsequent investigations.

### 3-(4,5-Dimethylthiazol-2-yl)-2,5-diphenyl-2H-tetrazolium bromide assay

3-(4,5-Dimethylthiazol-2-yl)-2,5-diphenyl-2H-tetrazolium bromide **(**MTT) assay was carried out to determine the effect of 3.5 mg/mL of SPIONs on the proliferation of hWJSCs. The cells were seeded in 96-well plates (5000 cells/200 μL), while medium change was undertaken after 24 h with either a solution of (1) complete culture medium (control group) or (2) SPIONs added to the culture medium. It should be mentioned that iron oxide [Chemical Abstracts Service (CAS) 1317-61-9: 13 wt%) and dextran (CAS 9004-54-0: 87 wt%)] were used based on the manufacturer’s protocol. SPIONs were used at a dose of 3.5 mg/mL based on a previous report [19]. The mentioned dose was shown to contain 327 μg/mL of iron based on atomic mass. After 24 h, 20 μL of a solution consisting of 5 mg/mL of MTT, a tetrazole (Sigma-Aldrich), was added to each well and incubated in an incubator with 5% CO_2_ and 100% humidity at 37°C for 4 h. The medium was later removed and, to dissolve the formazan crystals, 200 μL of DMSO was added per well (Merck). Cell viability was assessed at an optical density of 570 nm using a microplate reader (Floustar Omega, BMG Lab Tech, Ortenberg, Germany) (*n* = 4). The experiment was repeated three times for each sample.

### Prussian blue cell staining

Prussian blue staining was used to evaluate *in vitro* the efficiency of hWJSCs to uptake SPIONs. To do so, 10% formalin was used to fix hWJSCs. The cells were later washed three times with deionized water, and were then subjected to 2 mL of a solution consisting a 1:1 ratio of 20% aqueous solution of HCl (Merck) and 10% aqueous solution of potassium ferrocyanide (Sigma-Aldrich), and left at room temperature for 10 min. The cells were washed three times in distilled water and counterstained with nuclear fast red (Sigma-Aldrich) for 5 min, and finally rinsed twice in distilled water. A light microscope (Olympus, FSX100, Tokyo, Japan), xylazine and 95% ethanol (Merck) were used to visualize the SPIONs. Iron would be visible as bright blue, nuclei in red color and cytoplasm in pink.

### Preparation of ADM

Human skin samples that were aseptically provided by cosmetic surgeries in the Burn and Wound Healing Research Center, Department of Plastic Surgery, Shiraz University of Medical Sciences, Shiraz, Iran were used for preparation of ADM. Skin samples were transferred into a solution containing PBS, penicillin–streptomycin and fungisone. After removal of epidermis, fat tissue and hairs, the remaining tissue was washed with PBS (2x). The removal of epidermal tissue was undertaken using a solution containing 1 M NaCl, 0.5% Triton X-100 and 10 mol/L EDTA that was left in a shaking incubator at 37°C for 24 h. Decellularization was carried out in a solution containing 0.5% sodium dodecyl sulfate, 10 mmol/L HEPES (Sigma) and 10 mmol/L EDTA in an incubator at 37°C for 1 h. Then, the decellularized human skin sheet was cut into pieces 1.5 mm thick and 6 mm in diameter and lyophilized. To evaluate the scaffold, Hematoxylin and Eosin (H&E), Verhoff and Alcian blue staining methods were utilized for all samples. In summary, cell viability on ADM disks was determined on 96-well plates, while a cell population of 2.0 × 10^4^ cells/300 μL of DMEM was seeded on the top center of the ADM disks as previously described [8].

### Cell seeding

A total of 3.0 × 10^5^ hWJSCs seeded on ADM scaffold were incubated in an incubator with 5% CO_2_ at 37°C and saturated humidity for 24 h. Then, this scaffold seeded with cells was used for the third treatment group.

### Animals

Thirty male Sprague–Dawley rats (180–200 g) were obtained from the Laboratory Animal Center of Shiraz University of Medical Sciences, Shiraz, Iran. They were kept singly in cages at 22.0 ± 2.0°C and 12 h light/dark cycles. The animals had free access to food and water. Xylazine (20 mg/kg; Alfazyne) and ketamine (5 mg/kg; Woerden, The Netherlands) were injected intraperitoneally to anesthetize the animals before burn induction and treatment measures. Buprenorphine (0.05 mg/kg, Produlab Pharma) was injected subcutaneously three times daily to induce analgesia during interventions. Experiments were carried out based on the rules and regulations of the Iran Veterinary Organization. The study was financially supported and ethically approved by the National Institute for Medical Research Development of Iran Ministry of Health, Treatment and Education (Prof. Reza Malekzadeh, Dr. Ehsan Shamsi Gooshki, Dr. Abbas Mirshekari, and Dr. Mahmoud Motavasel Arani).

### Experimental design

The rats were randomly divided into three equal groups of 10. To induce a standard model of scald burn injury, 2.5 mL of boiling water (95°C) was transferred into a firm rubber ring (2 cm diameter) on the dorsal surface of the skin for 10 s. Then, the hot water was removed by sterile absorbent cotton wool. The control group did not receive treatment after burn induction. The second group was treated just by ADM scaffold located on the burn injury by two sutures, and the third group was cured similarly by ADM scaffold seeded with 3 × 10^5^ hWJSCs. The rats were euthanized after 1, 2 and 3 weeks post-intervention for tissue sampling. In each group, the rats were euthanized after 1 (3 rats), 2 (3 rats) and 3 (4 rats) weeks post-interventions for tissue sampling.

### Quantitative real-time polymerase chain reaction

Skin tissue samples were obtained from animals in three groups 7, 14 and 21 days following the interventions. The samples were 3 mm diameter sterile skin segments prepared using a cutter (Braun/Aesculap, Melsungen, Germany). Immediately after tissue samples were cut, they were frozen in liquid nitrogen and thereafter stored at −80°C until further use. The skin samples were harvested for total cellular RNA extraction 48 h after intervention using an RNA extraction kit (Cinna Gen Inc., Tehran, Iran) in order to quantify Bax and Bcl-2 gene expression. The quality and quantity of RNA were investigated by assessing the optical density ratio (A260/A280 and A260/A230) using a Nanodrop spectrophotometer (Nanodrop; Thermo Fisher Scientific,Waltham, USA). cDNA was synthesized from 1000 ng of total RNA in a first-strand cDNA synthesis reaction applying the Revert Aid first strand cDNA synthesis kit (Thermo Fisher Scientific, Waltham, USA). The Bax and Bcl-2 genes were used as target genes and B2m as an endogenous control gene. The sequences of genes of interest were determined using the National Center for Biotechnology Information (NCBI) database and primer sets were designed by primer3 software. Oligonucleotide sequences for 244 bp B2m, 134 bp Bcl-2 and 174 bp Bax were investigated (Table 2).

**Table 1:** Antibodies used in this study and their sources

**Name**	**Isotype**	**Fluorophore**	**Protein**	**Source**
Anti-CD34	IgG1	PE	Glycoprotein	Bio Legend
Anti-CD44	IgG2b	FITC	Glycoprotein	BD Biosciences
Anti-CD45	IgG2b	FITC	Receptor	Santa Cruz Biotechnology
Anti-CD73	IgG1	PE	5′-Nucleotidase	Bio Legend
Anti-CD90	IgG1	FITC	Glycoprotein	Bio Legend
Anti-CD105	IgG1	APC	Glycoprotein	BD Biosciences
Anti–Notch1	IgG1	APC	Receptor	eBioscience

**Table 2:** The Bax, Bcl-2 and B2m gene sequences designed by primer3 software

**Gene**	**Primer sequence**	**Size (bp)**
Bax	Forward: 5′-CTGCAGAGGATGATTGCTGA-3′ Reverse: 5′-GATCAGCTCGGGCACTTTAG-3′	174
Bcl-2	Forward: 5′-ATCGCTCTGTGGATGACTGAGTAC-3′ Reverse: 5′-AGAGACAGCCAGGAGAAATCAAAC-3′	134
B2m	Forward: 5′-**CGTGCTTGCCATTCAGAAA**-3′ Reverse: 5′**-ATATACATCGGTCTCGGTGG**-3′	244

**Table 3:** T2W MRI parameters including time of repetition (TR), time of echo (TE), slice thickness, matrix size and field of view (FOV) in both axial and sagittal views

**View**	**TR (ms)**	**TE (ms)**	**Slice thickness (mm)**	**Matrix size (mm)**	**FOV (mm)**
Axial	5840	97	1.5	256 × 147	180 × 129
Sagittal	2800	81	2	256 × 179	220 × 220

**Figure 1.**
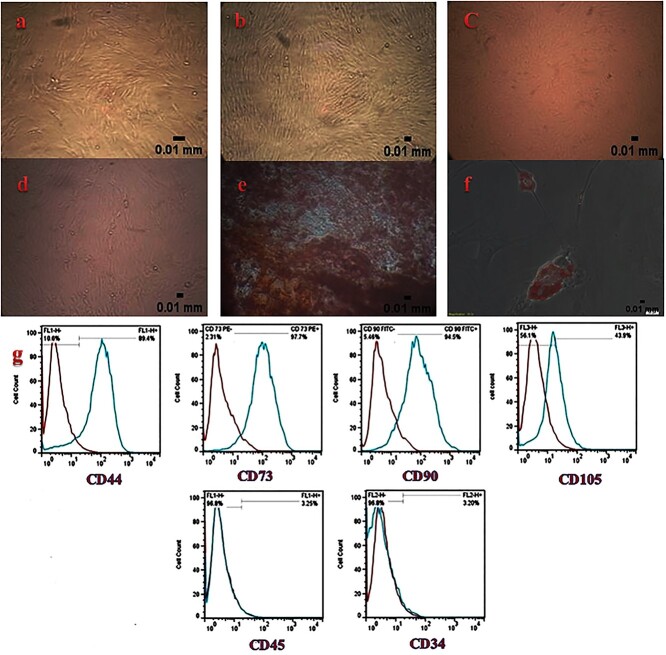
Characterization of human Wharton’s jelly stem cells: (**a**) passage 1 (20x), (**b**) passage 2 (20x), (**c**) passage 3 (20x), (**d**) passage 4 (20x), (**e**) osteogenic induction visualized in red color by alizarin red staining (40x), (**f**) adipogenic induction visualized in red color by Oil Red O staining (40x), (**g**) positive expression of mesenchymal stem cell markers for CD44, CD73, CD90 and CD105 and negative expression of hematopoietic stem cell markers for CD34 and CD45

Quantitative real-time polymerase chain reaction (PCR) was conducted with SYBR Green I as reporter dye and Step One Real-Time PCR reactions (Applied Biosystems, Waltham, USA). In each reaction, 200 nM of each primer was added to target the specific sequence. The PCR conditions were 10 min at 94°C followed by 40 cycles of 15 s at 94°C, 60 s at 60°C and melting curve analysis ramping from 65 to 95°C. The amplification signals of different samples were normalized to the B2m cycle threshold (Ct), and then to obtain the fold change in mRNA levels between various groups the DDCt method was used.

### Tensile testing

For tensile strength testing according to the ASTM D882 standard, the transplanted hWJSCs + ADM samples (5 × 1 cm^2^ rectangles surgically removed from the wound bed) were used. The samples were placed in a vacuum at ambient temperature and humidity and 25 mmHg pressure before the test. The Brookfield CT3 texture analyzer and a load cell of 4.5 kg were used for this test. To calculate the elongation at break (EB), the following equation was used, where *L*_0_ is the initial length of the sample and *L*_max_ is the maximum length at the break: $\mathrm{EB}\ (\%)=\frac{L_{max}}{L_0}\times 100$. Also, the following equation was used to calculate the ultimate tensile strength (UTS). In this equation, *F*_max_ is the maximum input load at tear point and *A* is the sample cross-section area: $\mathrm{UTS}=\frac{F_{max}}{A}$.

### Prussian blue histological staining

Histologic assessment was undertaken by fixation of tissue samples in 10% buffered formalin. They were later embedded in paraffin. Tissue sections of 4 μm thickness were provided and dried overnight at 37°C. Sections were deparaffinized, rehydrated with a graded ethanol series and finally stained with Prussian blue and visualized using an Olympus microscopic.

**Figure 2.**
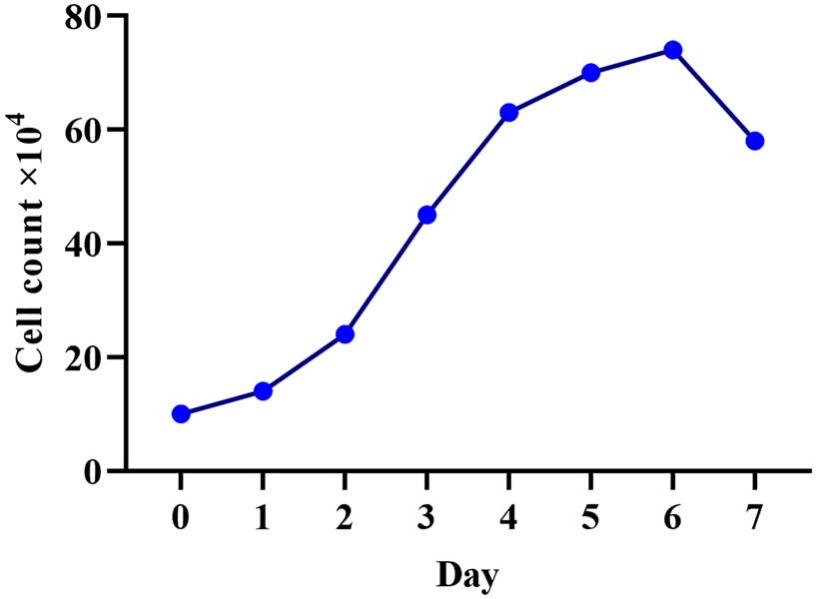
Growth kinetics of human Wharton’s jelly stem cells. Population doubling time of 40.1 h

**Figure 3.**
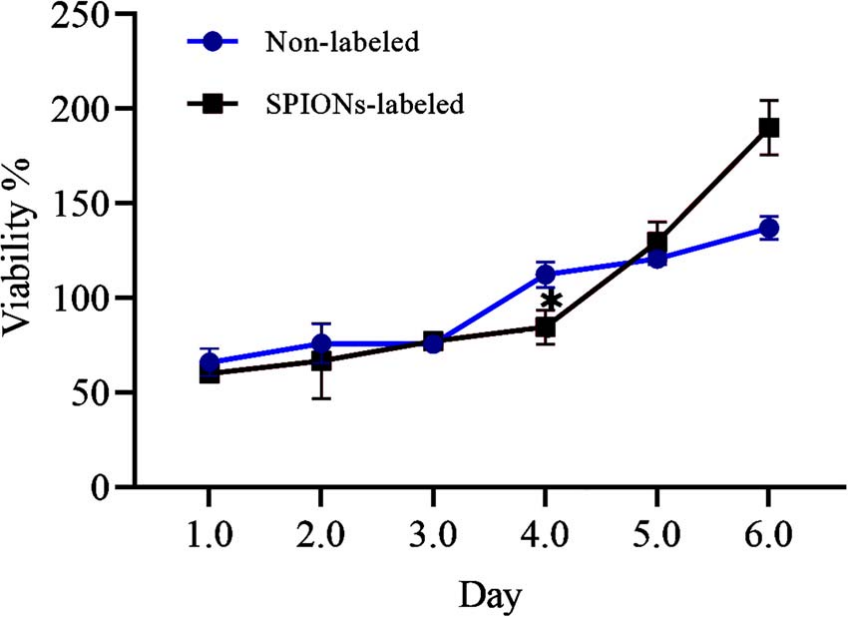
3-(4,5-Dimethylthiazol-2-yl)-2,5-diphenyl-2H-tetrazolium bromide (MTT) assay comparing the viability and proliferation of non-labeled and human Wharton’s jelly stem cells labeled with 3.5 mg/mL of superparamagnetic iron oxide nanoparticles (SPIONs) (^*^p = 0.01)

### Scanning electron microscopy

Tissue samples were fixed in phosphate buffer containing 2% glutaraldehyde for 12 h. They were later washed three times with a buffer solution of sodium cacodylate for 30 min each. Then, the samples were soaked in a solution of osmium tetroxide and 1% sodium cacodylate for 12 h and later dehydrated in a series of ethanol concentrations of 5, 10, 25, 50, 70 and 90%; samples were exposed to each concentration for 20 min. The samples were subjected to a final concentration of 100% ethanol together with CO_2_ to achieve the critical point drying of CO_2_ (CPD 7501 Critical Point Drier, polaron Range, Quorum Technologies Company Ltd, UK).

The samples were then deposited in gold ions in a pressure metallic chamber (III Desk, Denton Vacuum, LLC, Moorestown, NJ, USA) and were finally transferred for analysis by scanning electron microscopy (SEM) (JSM-6380LV, JEOL Ltd, Tokyo, Japan). SEM pictures were assessed using descriptive analysis of the topology and structural organization of the samples and by the presence, organization and density of collagen fibers in the ECM [25], using as parameters the uninjured tissue and neoformation analyzed from the burned tissue.

**Figure 4.**
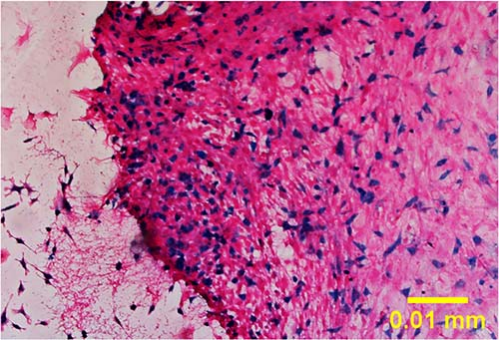
Prussian blue staining of internalization of superparamagnetic iron oxide nanoparticles within human Wharton’s jelly stem cells visualized in blue color (20x)

**Figure 5.**
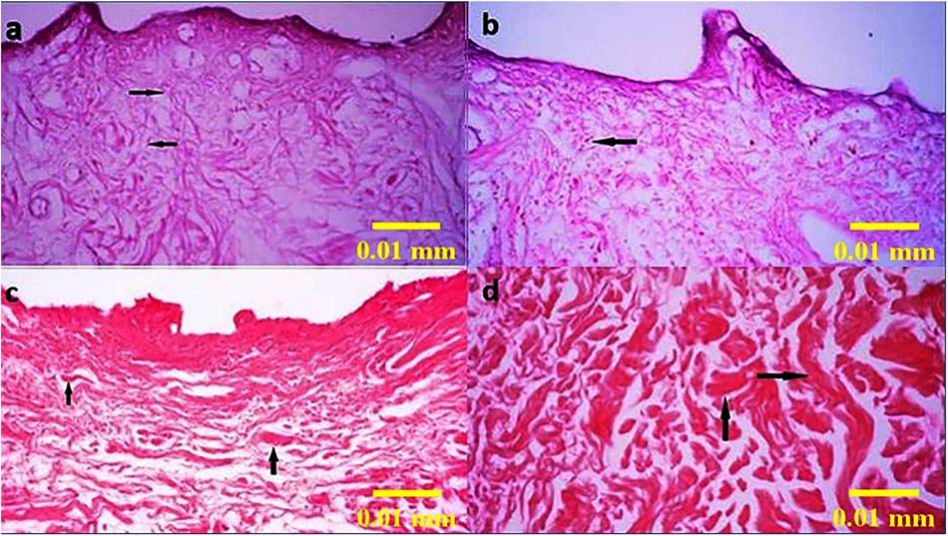
Histologic evaluation of the prepared acellular dermal matrix scaffold, before use in intervention groups 2 and 3, by the H&E staining method showing the absence of epidermis and presence of fibroblasts (**a** and **b**) and collagen fibers (**c** and **d**) demonstrated by black arrows (100×)

**Figure 6.**
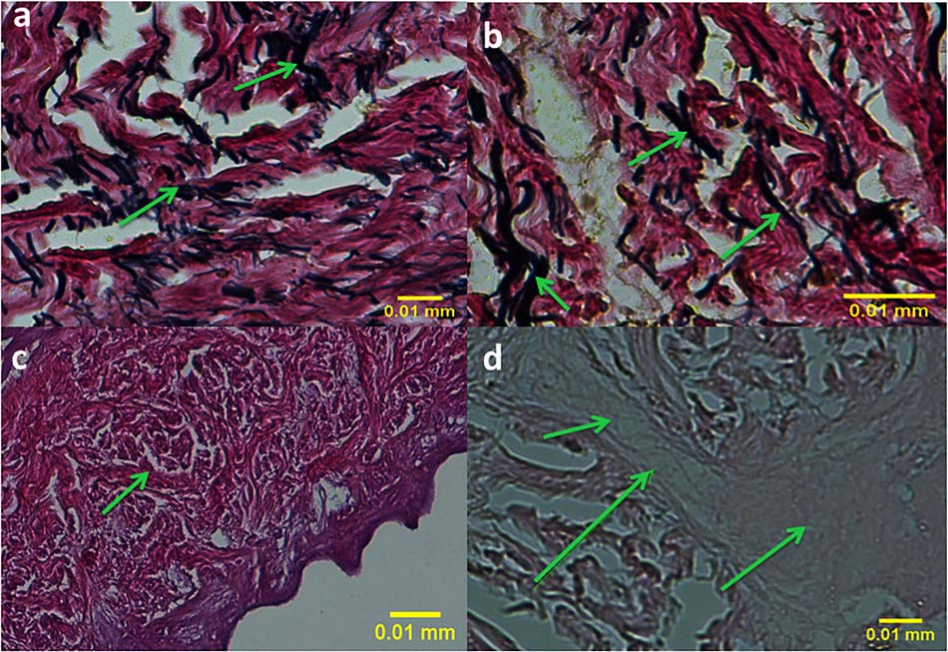
Histologic evaluation of prepared acellular dermal matrix scaffold samples, before use in intervention groups of 2 and 3, by the Verhoff staining method (**a** and **b**) showing the presence of elastic fibers in dark blue indicated by arrows and the Alcian blue staining method (**c** and **d**) showing the presence of glycosaminoglycans in light blue indicated by arrows (400×)

### Assessment by MRI

To track labeled cells with 2 × 10^6^ SPIONs *in vivo*, MRI was carried out for recipient rats 24 h, 7, 14 and 21 days after transplantation using a clinical 3.0 T MRI scanner (Siemens Syngo MR E11, 3.0 T, Germany). The T2-weighted MRI parameters are shown in Table 3. In the T2* image, the negative contrast, visualized as black, is related to the presence of iron oxide nanoparticles.

**Figure 7.**
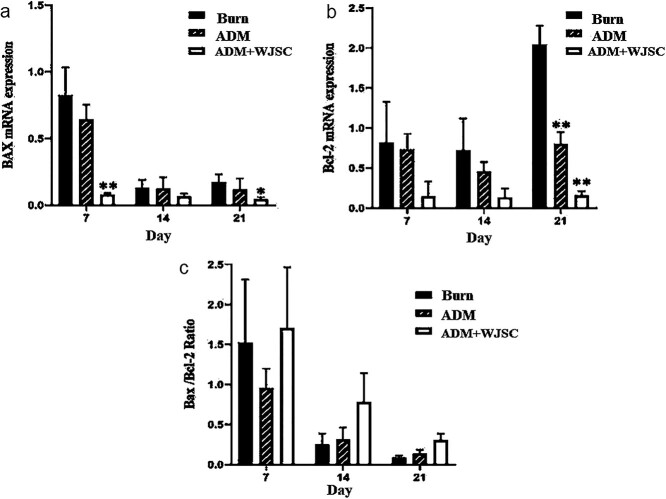
Comparing the expression of the Bax pro-apoptotic gene and Bcl-2 anti-apoptotic gene in group 3 [acellular dermal matrix (ADM) seeded with 3 × 10^5^ human Wharton’s jelly stem cells (hWJSCs) labeled with superparamagnetic iron oxide nanoparticles], with group 1 as burn control and group 2 treated with ADM alone. Tukey’s *post hoc* test was carried out to find out the difference between groups. Asterisks are used to show when a significant difference was detected between treatments (*p* = 0.01 for Bax and Bcl-2 genes). Expression data relative to those of the reference gene from at least three independent assays are given as mean ± standard deviation. Statistical significance was tested using two-way analysis of variance. *p < 0.05; **p < 0.01.

### Statistical analysis

The normal distribution of data was tested using the Kolmogorov–Smirnov test. The data sets were analyzed by one-way analysis of variance (ANOVA) and Prism software (GraphPad Software, version 6.0, San Diego, USA). There were 2-fold purposes for ANOVA to simultaneously compare more than two data sets. When a significant difference was detected between treatments, Tukey’s *post hoc* test was applied to find out which treatment differed from the others. A *p* value <0.05 was considered statistically significant.

**Figure 8.**
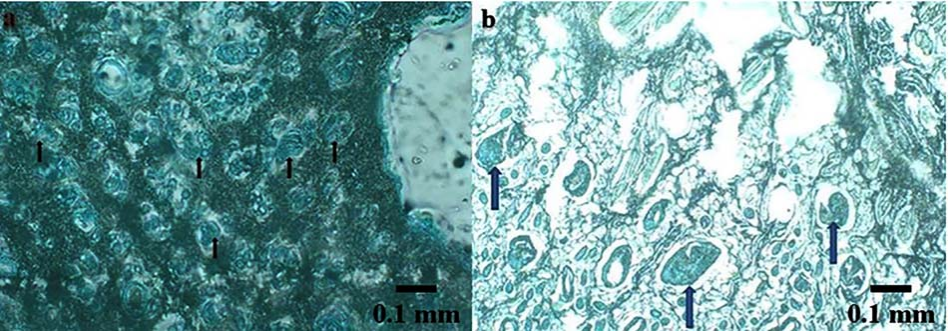
Histologic presence of superparamagnetic iron oxide nanoparticles can be seen in the tissue 21 days after burn induction and treatment with acellular dermal matrix seeded with human Wharton’s jelly stem cells (hWJSCs) labeled with superparamagnetic iron oxide nanoparticles (SPIONs) (**a**, **b**). Two animals in group 3 received acellular dermal matrix scaffold seeded with 3 × 10^5^ hWJSCs labeled with SPIONs. Prussian blue staining shows the presence of iron oxide nanoparticles (light blue, indicated by arrows) in two tissue samples; 40x

## Results

### Cell characterization

hWJSCs before and after labeling with SPIONs at different passages were adherent to the culture plates and were spindle shaped (Figure 1a–d). Regarding their osteogenic differentiation, hWJSCs exhibited calcium deposits in osteogenic medium after 3 weeks that was visualized as red color by alizarin red staining (Figure 1e). After adipogenic induction, hWJSCs stained with Oil Red-O revealed intracellular lipid droplets that were red in color (Figure 1f). These cells exhibited positive expression of CD44, CD73, CD90 and CD105 as mesenchymal markers and negative expression of CD34 and CD45 as hematopoietic markers (Figure 1g).

### Growth kinetics

hWJSCs after labeling with SPIONs at the third passage and culture for 7 days showed a PDT of 40.1 h. An increase in cell proliferation was noted until day 6, and then a decrease was noted (Figure 2). The experiment was repeated three times for each sample (*p* ≤ 0.05).

**Figure 9.**
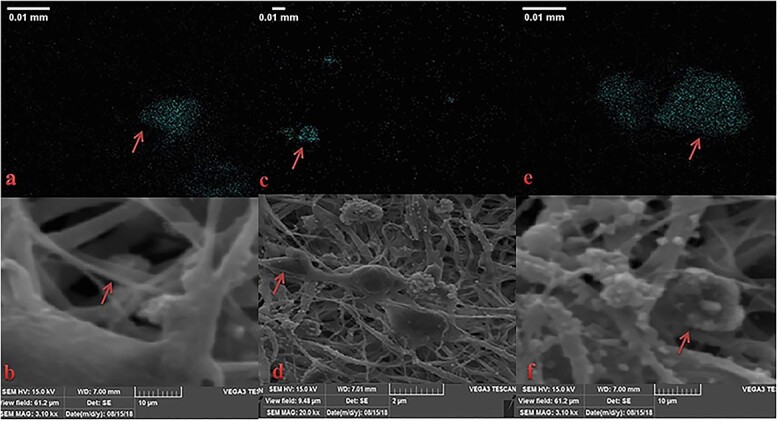
Scanning electron microscopy images of burn wound treated with acellular dermal matrix (ADM) scaffold seeded with 3 × 10^5^ human Wharton’s jelly stem cells (hWJSCs) labeled with superparamagnetic iron oxide nanoparticles (SPIONs) (**a**–**f**). The presence of iron oxide nanoparticles in the organized extracellular matrix structure is seen in blue after 21 days (arrows) in three tissue samples of group 3 treated with ADM seeded with WJSCs labeled with SPIONs; magnification of T20000

### MTT assay

MTT assay did not reveal any reduction in proliferation of labeled hWJSCs with 3.5 mg/mL of SPIONs in comparison to non-labeled cells until day 6. A positive effect on viability and proliferation of hWJSCs was demonstrated on day 6 after addition of SPIONs to the medium for cell labeling, when compared to non-labeled cells (Figure 3). The experiment was repeated three times for each sample (*p* = 0.01).

### Prussian blue cell staining

Internalization of SPIONs within hWJSCs in blue color was shown after Prussian blue staining (Figure 4).

### Histological evaluation of ADM

#### H&E staining of ADM

Using the H&E staining method, the prepared ADM samples were assessed. Figure 5 shows the absence of epidermis and decellularization in the dermal tissue of prepared ADM samples that were further used in intervention groups 2 and 3.

#### Verhoff and Alcian blue staining of ADM

Using Verhoff and Alcian blue staining methods, ADM samples revealed the presence of elastin fibers in ADM scaffold, in blue color, and glycosaminoglycans (Figure 6).

### Quantitative real-time PCR

The quantitative real-time PCR findings revealed a significant decrease in the expression of the Bcl-2 anti-apoptotic gene in the group receiving just ADM and labeled hWJSCs seeded on ADM at 21 days (*p* < 0.05). Also, expression of the pro-apoptotic gene Bax significantly decreased in the groups with labeled cells seeded on ADM and ADM alone (*p* < 0.05). The Bax/Bcl-2 ratio, which is one of the best markers of apoptosis, was also examined at different time points; despite the increase in this index in the ADM + WJSCs group, no significant relationship was seen (Figure 7).

### Tensile testing

The mechanical properties of the burn skin wounds treated with labeled hWJSCs seeded onto ADM compared with normal skin after 21 days were acceptable (Table 4).

### Histological assessment of SPIONs

The presence of SPIONs in the tissue, 3 weeks after burn injury and treatment with hWJSCs labeled with SPIONs and seeded onto ADM, is shown by the presence of iron oxide inside the cells seen as light blue color when stained by Prussian blue (Figure 8).

**Table 4:** Mechanical properties of the acellular dermal matrix and human skin

**Sample**	**Ultimate tensile strength (MPa)**	**Young’s modulus** **(MPa)**
ADM (*n* = 10)	9.08$\pm$0.87	41.09$\pm$6.50
Normal skin (*n* = 10)	0.10–57	0.26–141

### SEM

The SEM technique used in this study was very efficient for displaying the distribution of SPIONs in hWJSCs seeded on ADM after burn wounds with good resolution; iron core clusters are visible as light blue color (Figure 9).

**Figure 10.**
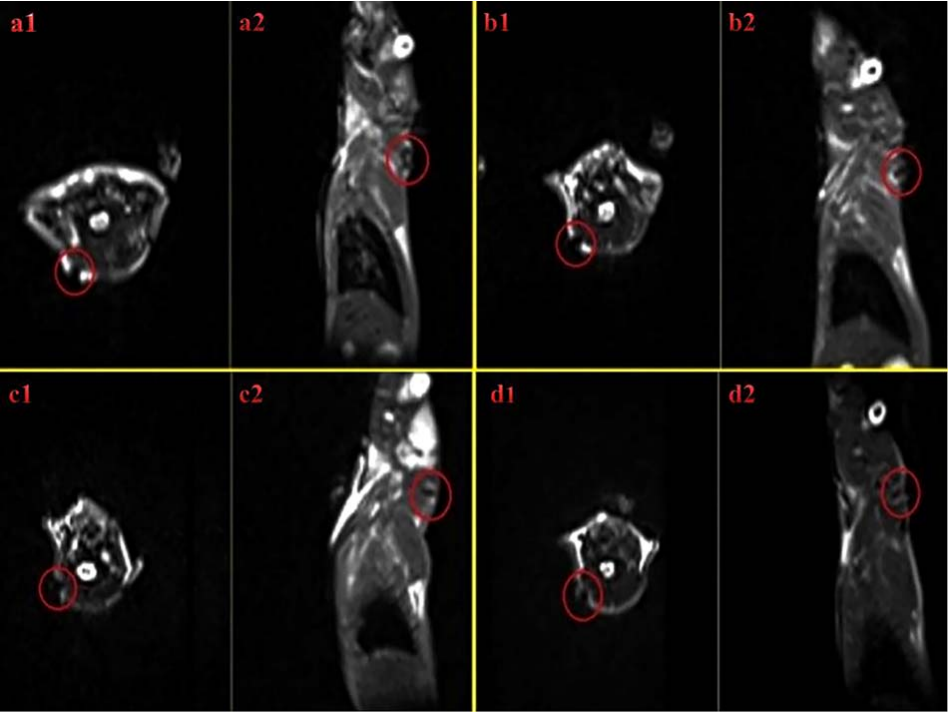
T2W images of injected rat with human Wharton’s jelly stem cells (hWJSCs) labeled with superparamagnetic iron oxide nanoparticles (SPIONs) within axial and sagittal views. Axial and sagittal views are shown: (**a1** and **a2**) immediately after injection, (**b1** and **b2**) 24 h after injection, (**c1** and **c2**) a week after injection, and (**d1** and **d2**) 2 weeks after injection. The signal related to control H_2_O in T2* imaging is seen in white. The thin black rim denotes the borders of the container that lacks any signal. Red circles indicate the negative contrast of iron oxide nanoparticles in group 3 treated with acellular dermal matrix seeded with 3 × 10^5^ hWJSCs labeled with SPIONs. In the T2* image, the negative contrast is seen as black and is related to the presence of iron oxide nanoparticles

### *In vivo* assessment by MRI

T2W images of injected rats with WJSCs labeled with SPIONs in axial and sagittal views are shown in Figure 10. Red circles indicate the negative contrast of iron oxide nanoparticles. There was no sign of negative contrast in axial images, where no iron oxide was present.

## Discussion

Burn still has devastating effects both functionally and cosmetically on the affected patients necessitating the development of more efficient treatment methods [26–27]. MSCs have recently been used in this field to promote superior healing of burn wounds better and faster, as they can decrease the inflammatory process [8]. Transplantation of stem cells in the healing of burn wounds dates back to 2003 when the use of bone marrow-derived stem cells (BMSCs) was compared to embryonic fibroblasts [28]. In this study, MRI-tracking of hWJSCs labeled with SPIONs and seeded onto ADM in the healing of burn wounds was investigated. First, hWJSCs were characterized and found to have MSC properties, and their growth kinetics were found to show similar results to a previous histological study assessing the use of hWJSCs in the healing of burn injuries [8]. Tissue engineering utilizing scaffolds together with cell therapy opened a window into the treatment of burn injuries, resulting in faster and more effective wound healing [29]. An attractive strategy is to use biocompatible materials to replace inadequate healthy skin of the patients [30].

The results of the MTT assay in our study confirmed the absence of any decrease in proliferation of labeled hWJSCs with 3.5 mg/mL of SPIONs in comparison to non-labeled cells until day 6. In fact, a positive effect on viability and proliferation of hWJSCs was demonstrated on day 6 after the addition of SPIONs to the cell labeling medium, when compared to non-labeled cells. In another study, exposure to SPIONs for over 96 h was shown not to decrease cell survival and differentiation [31]. Regarding the role of magnetic nanoparticles in stem cells, Wu *et al*. reported that magnetic nanoparticles with BMSCs can improve angiogenesis and fibroblast function and proliferation and as a result enhance wound healing [32]. In scald burns of rats, topical application of BMSCs together with fibrin glue was also shown to participate in healing [33]. Leonardi *et al*. applied BMSCs in artificial dermal substitutes to improve wound healing [34].

ADM as a natural promising dermal scaffold derived from allogeneic or heterogeneous skin sources has successfully been used in tissue repair and improvement of cell colonization and combined favorably with the wound site edge [35,36]. Construction and histological characterization of ADM and hWJSCs was performed in our study. Qi *et al*. using ADM seeded with BMSCs reported significant wound healing in mice that is identical to our findings [37]. Nazempour *et al*. seeded WJSCs onto ADM scaffold and demonstrated improved angiogenesis and granulation tissue formation and decreased inflammation, necrosis and fibrosis after 21 days [8]. Orbay *et al*. found that adipose tissue derived stem cells seeded onto ADM could promote wound vascularization, volume maintenance and collagen quantity [38]. Formigli *et al*. illustrated that MSCs seeded on bioengineered scaffolds could improve healing of skin wounds in rats [39]. The healing effect of MSCs in burns can be due to their anti-inflammatory role in the upregulation of anti-inflammatory markers, downregulation of inflammatory markers and a local increase in anti-inflammatory cytokines [40]. They also have a vital immunomodulatory role due to secretion of ‘secretomes’ or extracellular vesicles that can downregulate interleukin (IL)-6 and nitric oxide synthase and increase IL-10 and ATP [41].

Despite significant progress in regenerative medicine, cell tracking after transplantation remains elusive due to the lack of effective, non-invasive, non-toxic and clinically acceptable methods. MRI has been introduced as a non-invasive efficient method with high resolution to monitor migration and integration of cells after transplantation, using SPIONs as contrast magnetic agents to be distinguished by MRI [42]. The ability of MRI to track cells can be enhanced by labelling the cells with dextran-coated SPIONS [43]. We observed SPIONs to be taken up by hWJSCs through endocytosis and successfully illustrated that hWJSCs labeled with dextran-coated SPIONs could efficiently be detected and traced by MRI. In our study, the size of the SPIONs was 130 nm and a 3.0 T MRI was used. There are several studies using 1.5 T and 3 T MRI scanners [44,45] that were shown to yield the greatest contrast to trace and visualize labeled cells in hepatocellular carcinoma [46]. Transplantation of SPION-labeled MSCs in rats via the portal veins could be visualized by MRI and indicated an improvement in liver cirrhosis [47]. MRI scans demonstrated the production of hypointense signals in L-lysine-functionalized magnetic iron oxide nanoparticle (lys-IONP)-labelled BMSCs with improved signal intensity and minimum loss over 28 days, illustrating the use of lys-IONPs as long-term stem cell labeling and imaging agents [48].

Lee *et al*. utilized different endocytosis inhibitors and recognized the potential cellular internalization pathway of SPIONs in clathrin-mediated endocytosis, demonstrating that MSCs can act as effective nanoparticle carriers ensuring the successful localization of magnetic particles [31]. MRI has also successfully been utilized for tracking of SPION-labeled MSCs injected into the renal arteries and portal veins without any hazardous effect on the viability and differentiation of the transplanted cells [49]. MSCs labeled with MRI-visible nanoparticles could be used to visualize the reduction of a stroke infarct following cell transplantation [50]. *In vivo* MRI of labeled cells in osteochondral defects using magnetic nanoparticles with a diameter between 11 and 150 nm was previously shown [51]. Lee *et al*. successfully used MRI to track ferumoxytol-labeled MSCs [52]. These studies confirm our successful tracking of hWJSCS after seeding onto ADM and transplantation in burn wounds.

Zare *et al*. reported the *in vitro* and *in vivo* safety of MRI in tracking dental pulp stem cells (DPSCs) labeled with SPIONs without any significant reduction in the viability, proliferation and differentiation properties of the labeled cells, and demonstrated the internalization of SPIONs within DPSCs not to be toxic for the stem cells. An MRI-based method was demonstrated to successfully monitor DPSCs labeled with dextran-coated SPIONs without any significant effect on the stemness of DPSCs [19]. Xie *et al*. investigated the safety of MRI in the tracking of labeled MSCs surgically transplanted into a rat spinal cord segment, indicating that MSCs could be visualized by MRI *in vivo* with comprehensive safety [53]. Shahor *et al*. reported the safety of MRI in tracing MSCs labeled with SPIONs and as a non-invasive method in a traumatic brain injury murine model [54].

We also confirmed incorporation of hWJSCs labeled with SPIONs by Prussian blue staining and SEM. High efficiency of Prussian blue staining for internalization of SPIONs in MSCs has been shown previously [19,55]. SPIONs have little or no negative effect on viability, proliferation and function of cells, making them valuable for cell therapy purposes [56]. Our findings also indicated a decrease in apoptosis and an increase in Bcl-2 expression when SPION-labeled cells seeded onto ADM were used for healing of burn injuries. In our study, SPIONs did not induce any cytotoxicity in hWJSCs used for cell transplantation. After 21 days, comparison of the mechanical properties of the normal skin with burn wounds treated with labeled hWJSCs and seeded onto ADM demonstrated acceptable mechanical properties and healing, with relatively regular orientation of collagen fibers. Qi *et al*., using SEM assessment of burn wounds after cell transplantation and seeding onto ADM, demonstrated that collagen fibers were unevenly thickened, but had relatively regular orientation and maintained a porous network structure [37].

Several limitations were present in our study, such as use of an experimental burn injury rat model as a model of burn wounds in humans. While histologic structures and healing characteristics are different in rats and humans, contraction is an important part of wound healing in rats and re-epithelialization is its major counterpart in humans. Also MRI tracking in rats was not easily affordable that became possible by several practices.

## Conclusions

MRI was confirmed by Prussian blue staining and SEM to be a non-invasive and safe method of tracking transplanted cells labeled with SPIONs in burn wounds. Our findings open a new window into regenerative medicine for burn injuries in which MRI is targeted to track nanoparticles in labeled transplanted cells. Also, further investigation post-SPION labeling may need to be conducted to compare labeled and non-labeled hWJSCs for genomic instability and any probable up-regulation of genes related to tumorigenicity.

## Abbreviations

ADM: Acellular dermal matrix; ANOVA: Analysis of variance; BMSC: Bone marrow-derived stem cell; CD: Cluster of differentiation; DMEM: Dulbecco’s modified Eagle’s medium; DPSC: Dental pulp stem cell; ECM: Extracellular matrix; EB: Elongation at break; hWJSCs: Human Wharton’s jelly stem cells; MRI: Magnetic resonance imaging; MSC: Mesenchymal stem cells; PDT: Population doubling time; SPIONs: Superparamagnetic iron oxide nanoparticles; SEM: Scanning electron microscopy; UTS: Ultimate tensile strength.

## Data Availability

The data associated with this manuscript is available upon request to the corresponding author or the senior author.
